# Reactions of *Nicotiana *species to inoculation with monopartite and bipartite begomoviruses

**DOI:** 10.1186/1743-422X-8-475

**Published:** 2011-10-19

**Authors:** Sohail Akhtar, Rob W Briddon, Shahid Mansoor

**Affiliations:** 1 Agricultural Biotechnology Division, National Institute for Biotechnology and Genetic Engineering, Faisalabad, Pakistan

## Abstract

**Background:**

Some *Nicotiana *species are widely used as experimental hosts for plant viruses. *Nicotiana *species differ in ploidy levels, chromosome numbers and have diverse geographical origins. Thus, these species are useful model systems to investigate virus-host interactions, co-evolution of pathogens and hosts and the effects of ploidy level on virus resistance/susceptibility.

**Results:**

Here we have studied the responses of seven *Nicotiana *species to inoculation with *Cotton leaf curl Multan virus *(CLCuMV), a monopartite begomovirus, and *Tomato leaf curl New Delhi virus *(ToLCNDV), a bipartite begomovirus, both from the Indian subcontinent. All *Nicotiana *species supported the replication of both begomoviruses in inoculated leaves. However, only three *Nicotiana *species, namely *N. benthamiana*, *N. tabacum *and *N. sylvestris *showed symptoms when inoculated with ToLCNDV, while *N. benthamiana *was the only species that developed leaf curl symptoms when inoculated with CLCuMV. CLCuMV accumulated to detectable levels in *N. tabacum*, but plants remained asymptomatic. A previously identified mutation of RNA dependent RNA polymerase 1 was shown to be present only in *N. benthamiana*. The finding is in line with earlier results showing that the susceptibility of this species to a diverse range of plant viruses correlates with a defective RNA silencing-mediated host defense.

**Conclusions:**

The results presented show that individual *Nicotiana *species respond differently to inoculation with begomoviruses. The inability of begomoviruses to systemically infect several *Nicotiana *species is likely due to inhibition of virus movement, rather than replication, and thus provides a novel model to study virus-host interactions in resistant/susceptible hosts.

## Background

The genus *Nicotiana *(family *Solanaceae*) comprises 77 species, of which 40 are diploid and 37 allopolyploids. Named after the French ambassador to Portugal, Jean Nicot, who introduced tobacco to France in 1559, it is believed to have evolved in South America and then dispersed to Australia, Africa and southwestern North America [1]. The genus has been the focus of intense study which has provided information concerning the evolutionary relationships among different species found in this genus. Notably, *Nicotiana benthamiana *and *N. tabacum *(both allotetraploid) have become species used extensively in cytogenetic and plant virology studies [2].

Geminiviruses are single-stranded (ss)DNA viruses with circular genomes and are classified into four genera based on host range, insect vector and genome organization [3]. They are widely distributed throughout the world and infect either monocotyledonous or dicotyledonous hosts. All geminiviruses that infect monocotyledonous plants belong to the genus *Mastrevirus*, have genomes consisting of a single component and are transmitted by leafhoppers. A small number of dicot-infecting mastreviruses have also been identified. Viruses of the genus *Curtovirus *have single component genomes and are transmitted by leafhoppers. The genus *Topocuvirus *encompasses a single species with a genome consisting of a single component and is transmitted by treehopper. Viruses of the genus *Begomovirus *are transmitted by a single species of whitefly, *Bemisia tabac*i, and have genomes that consist of either a single ssDNA or two ssDNA components. The two components of bipartite begomoviruses are referred to as DNA A and DNA B, and both are, for most species, essential for symptomatic infection of plants. Monopartite begomoviruses are often associated with DNA satellites known as alphasatellites and betasatellites [4]. These begomovirus disease complexes are widespread in the Old World and constitute the largest group of begomoviruses. Studies on begomoviruses and their associated satellites suggest that they co-evolved with their hosts [5].

RNA silencing is an antiviral defense mechanism of plants that is induced by the replication of viruses and formation of viral double-stranded (ds)RNAs. These dsRNAs are the precursors of small interfering (si)RNAs that are involved in RNA silencing pathways [6-8]. Plant viruses counteract these defenses by encoding suppressors of gene silencing. Depending on the virus, individual viral proteins with differing primary functions in viral infection act as suppressors, interfering with distinct steps of the silencing pathway [9]. Various proteins encoded by begomoviruses and their satellites have been reported as suppressors of gene silencing; such as βC1 encoded by the betasatellite, which is the major symptom determinant encoded by begomovirus disease complexes [10,11]. Suppressors of RNA silencing encoded by viruses may influence micro (mi)RNA levels and thus may be responsible for the symptoms induced by infection. miRNAs are involved in the control of gene expression during growth and development of plants [12].

The study reported here has analysed the infectivity of monopartite and bipartite begomoviruses to various diploid and tetraploid *Nicotiana *species so as to assess the effects of ploidy level on susceptibility to begomoviruses. Local as well as systemic infection was determined by using PCR and Southern hybridization techniques. We tried to observe the presence of RDR1m in all the species and especially in those which are susceptible to viruses used in experiments. We also tried to link the origin of species and evolution of geminiviruses in different geographical locations.

## Results

### Infectivity of Cotton leaf curl Multan virus to *Nicotiana *species

**Figure 1:**
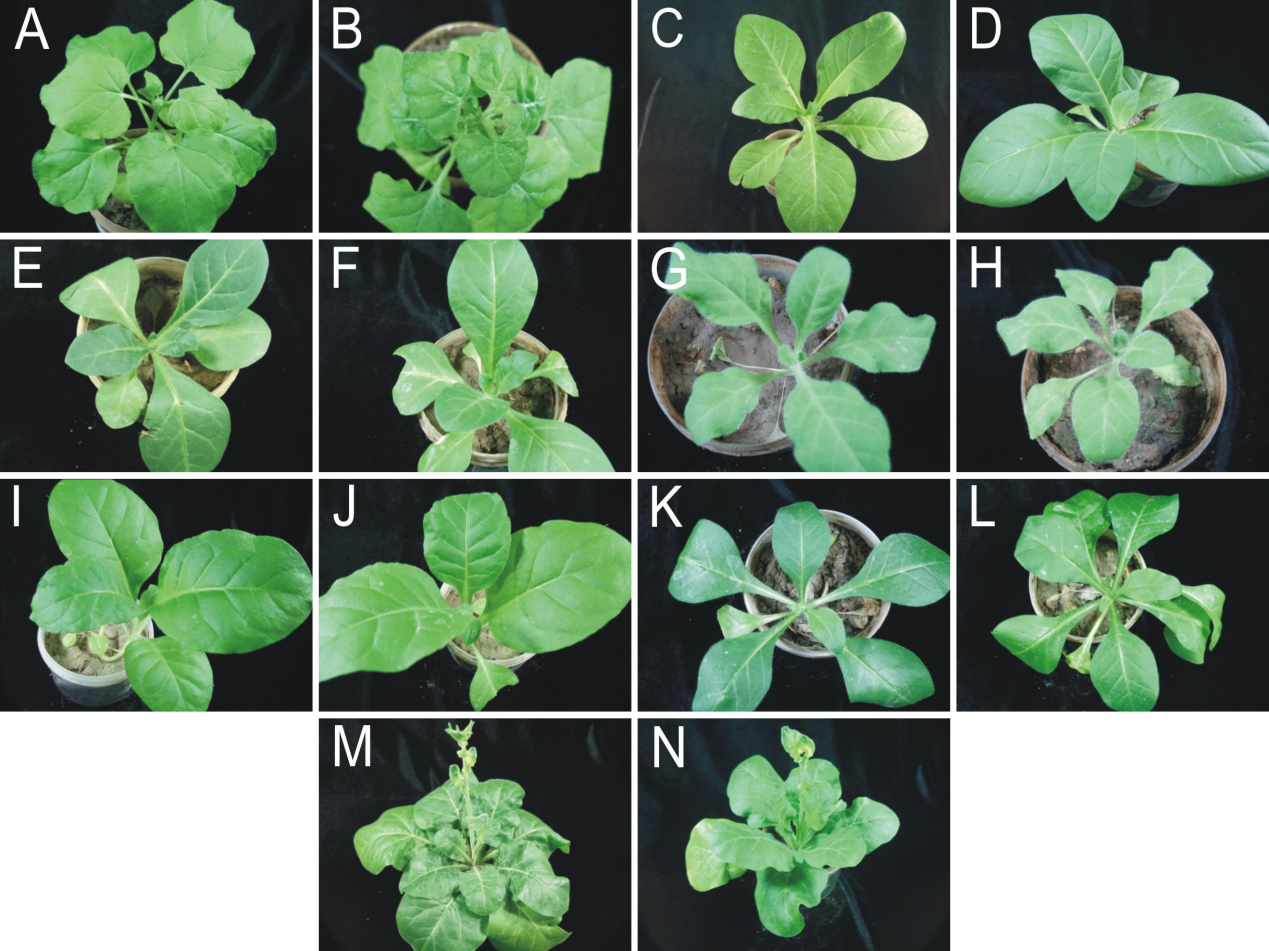
**Photographs of *Nicotiana *species at 25 days after inoculation with CLCuMV**. Shown are photographs of non-inoculated *N. benthamiana *(A), *N. sylvestris *(C), *N. nudicaulis *(E), *N. obtusifolia *(G), *N. tabacum *(I), *N. suaveolens *(K) and *N. repanda *(M) plants and photographs of *N. benthamiana *(B), *N. sylvestris *(D), *N. nudicaulis *(F), *N. obtusifolia *(H), *N. tabacum *(J), *N. suaveolens *(L) and *N. repanda *(N) plants inoculated with CLCuMV.

**Figure 2:**
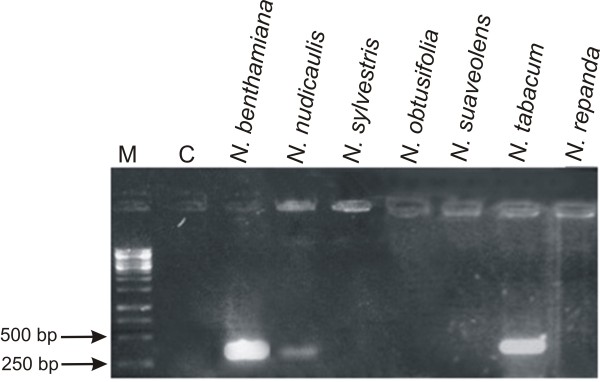
**PCR-mediated detection of CLCuMV in inoculated *Nicotiana *plants**. The ethidium bromide-stained agarose gel was photographed under UV illumination. The samples loaded on the gel resulted from PCR reactions with primer pair PK3AV2F/PK3AV2R and DNA extracted from the leaves of plants (as indicated above each well) inoculated with CLCuMV. The leaves sampled were developing at the time of, or developed after, inoculation and were sampled at 25 dpi. The presence of a 311 bp band indicates the systemic movement of CLCuMV from the site of inoculation. The sample in lane C resulted from PCR amplification with DNA extracted from a healthy *N. benthamiana *plant. A DNA size marker was electrophoresed in lane M. The sizes (bp) of selected marker bands are indicated on the left.

**Figure 3:**
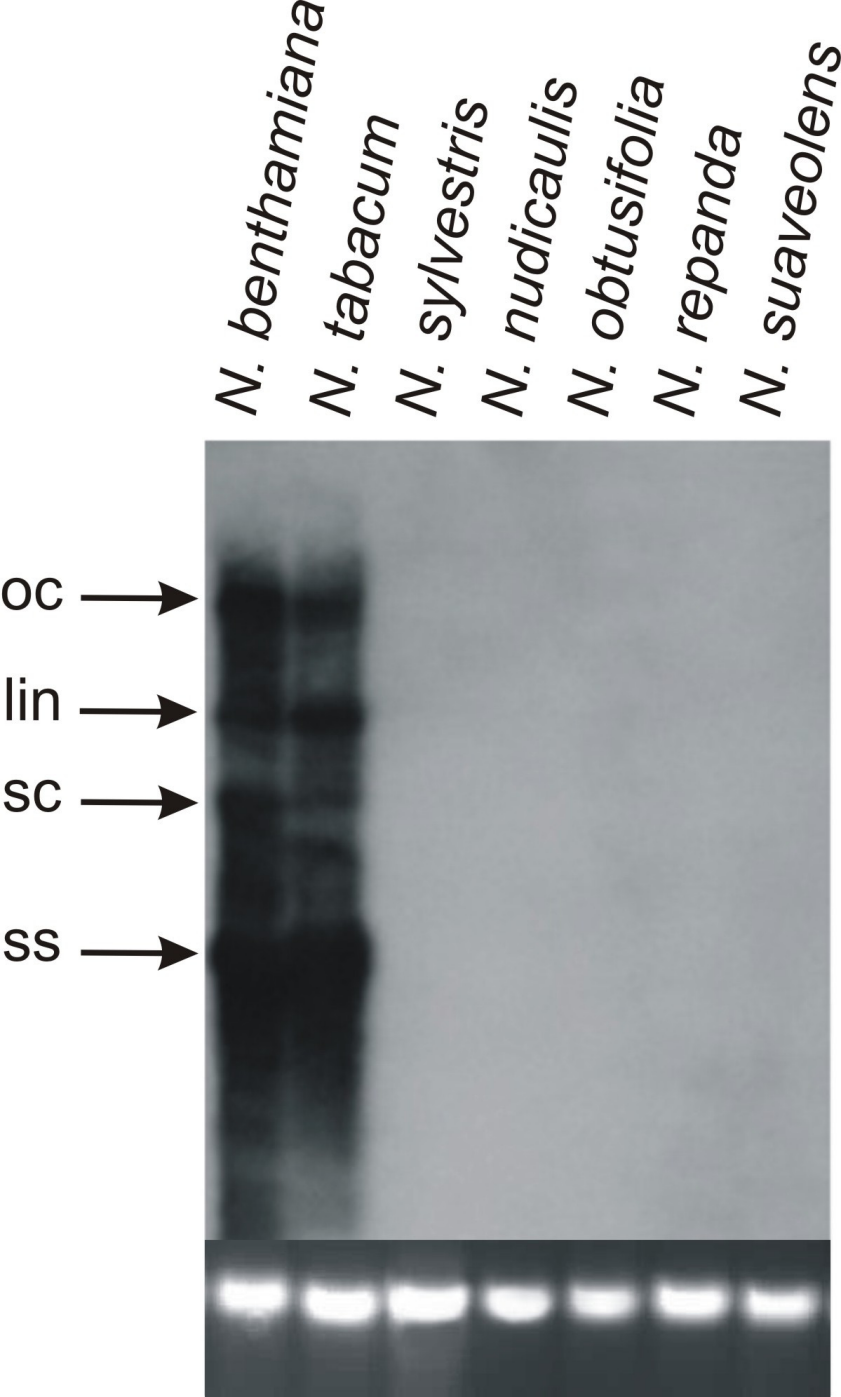
**Southern blot detection of CLCuMV in inoculated *Nicotiana *plants**. The DNA samples loaded on the gel were extracted from leaves of plants (as indicated above each well) inoculated with CLCuMV. The leaves sampled were developing at the time of, or developed after, inoculation and were sampled at 25 dpi. An approximately equal amount of DNA (10 μg) was loaded in each case. The blot was probed with a DIG-labeled CLCuMV fragment. The positions of replicative forms of viral DNA are indicated as open circular (oc), linear (lin), super coiled (sc) and single stranded (ss). A photograph of the genomic DNA bands on the ethidium bromide stained agarose gel to confirm equal loading is shown at the base.

To assess the ability of begomoviruses to replicate and move systemically from site of inoculation, infectious clones of representative monopartite and bipartite begomoviruses were introduced into plants by agroinfiltration [13,14]. Inoculation with the monopartite begomovirus *Cotton leaf curl Multan virus *(CLCuMV) led to characteristic leaf curl symptoms only in *N. benthamiana *plants at 25 days post-inoculation (Table 1). Plants of all the other species investigated remained symptomless (Figure 1; Table 1, 2). Total genomic DNA was extracted from inoculated leaves, as well as leaves developing subsequent to inoculation, for PCR and Southern hybridization to assess virus replication and movement. PCR results showed that in *N. benthamiana *and *N. tabacum *CLCuMV was able to spread from the site of inoculation (Figure 2). A very low-level amplification in case of *N. nudicaulis *indicated poor movement of the virus in this species. For the remaining *Nicotiana *species no viral DNA was detected by PCR in upper leaves, indicative of a lack of virus movement from the site of inoculation (Figure 2). A Southern blot of DNA extracted from leaves developing subsequent to inoculation probed for the presence of viral DNA showed a high virus titre in *N. benthamiana *and *N. tabacum *(Figure 3; Table 2). However, for *N. nudicaulis*, which was virus positive by PCR, no viral DNA was detected in the upper leaves. This suggests that virus levels in *N. nudicaulis *were very low, below the detection threshold of Southern hybridization

**Table 1:** Summary of the results of the infectivity studies

Species (ploidy level, chromosome number)	Parents	Origin	Infectivity of ToLCNDV (plants symptomatic/plants inoculated)	Plants PCR positive for ToLCNDV with primers ToLCNDVV2F/ToLCNDVV2R	Infectivity of CLCuMV (plants symptomatic/plants inoculated)	Plants PCR positive for CLCuMV with primers PK3AV2F/PK3AV2R	Plants PCR positive for CLCuMV with primers BegomoF/BegomoR
*N. sylvestris *(2x = 24)	-	South America	4/4	4	0/4	0	0 (0.7)*
*N. obtusifolia *(2x = 24)	-	Southwestern USA, Mexico	0/4	0	0/4	0	0 (1.4)*
*N. benthamiana *(4x = 38)	*N. sylvestris (likely maternal parent)*	Australia	4/4	4	4/4	4	4 (2.8)*
*N. suaveolens *(4x = 32)	*N. sylvestris (likely maternal parent)*	Australia	0/4	0	0/4	0	0
*N. tabacum *(4x = 48)	*N. tomentosiformis, N. sylvestris*	Eastern North America	4/4	4	0/4	4	4 (2.8)*
*N. nudicaulis *(4x = 48)	*N. obtusifolia, N. sylvestris*	North America	0/4	4	0/4	2	2 (2.8)*
*N. repanda *(4x = 48)	*N. obtusifolia, N. sylvestris*	Southern Texas (USA)	0/4	4	0/4	0	0 (1.4)*

*The sizes of bands produced in PCR reactions with primers BegomoF/BegomoR are shown in kb. The expected size product is 2.8kb for a full CLCuMV genome.

**Table 2:** Summary of Southern hybridization results for the detection of CLCuMV and ToLCNDV in inoculated *Nicotiana *plants

*Nicotiana *species	CLCuMV		ToLCNDV
	
	**Replication**^#^	Movement^*^	Movement*
*N. benthamiana*	+	+	+
*N. tabacum*	+	+	+
*N. sylvestris*	+	-	+
*N. obtusifolia*	+	-	-
*N. nudicaulis*	+	-	+
*N. suaveolens*	+	-	-
*N. repanda*	+	-	-

Hybridization is indicated as either positive (+), indicating the presence of bands hybridizing to the probe, or negative (-), no bands hybridizing to the probe.

*Detection of hybridizing bands in DNA extracted from leaves developing at the time of, or subsequent to, inoculation.

^#^Detection of hybridizing bands in inoculated leaves.

**Figure 4:**
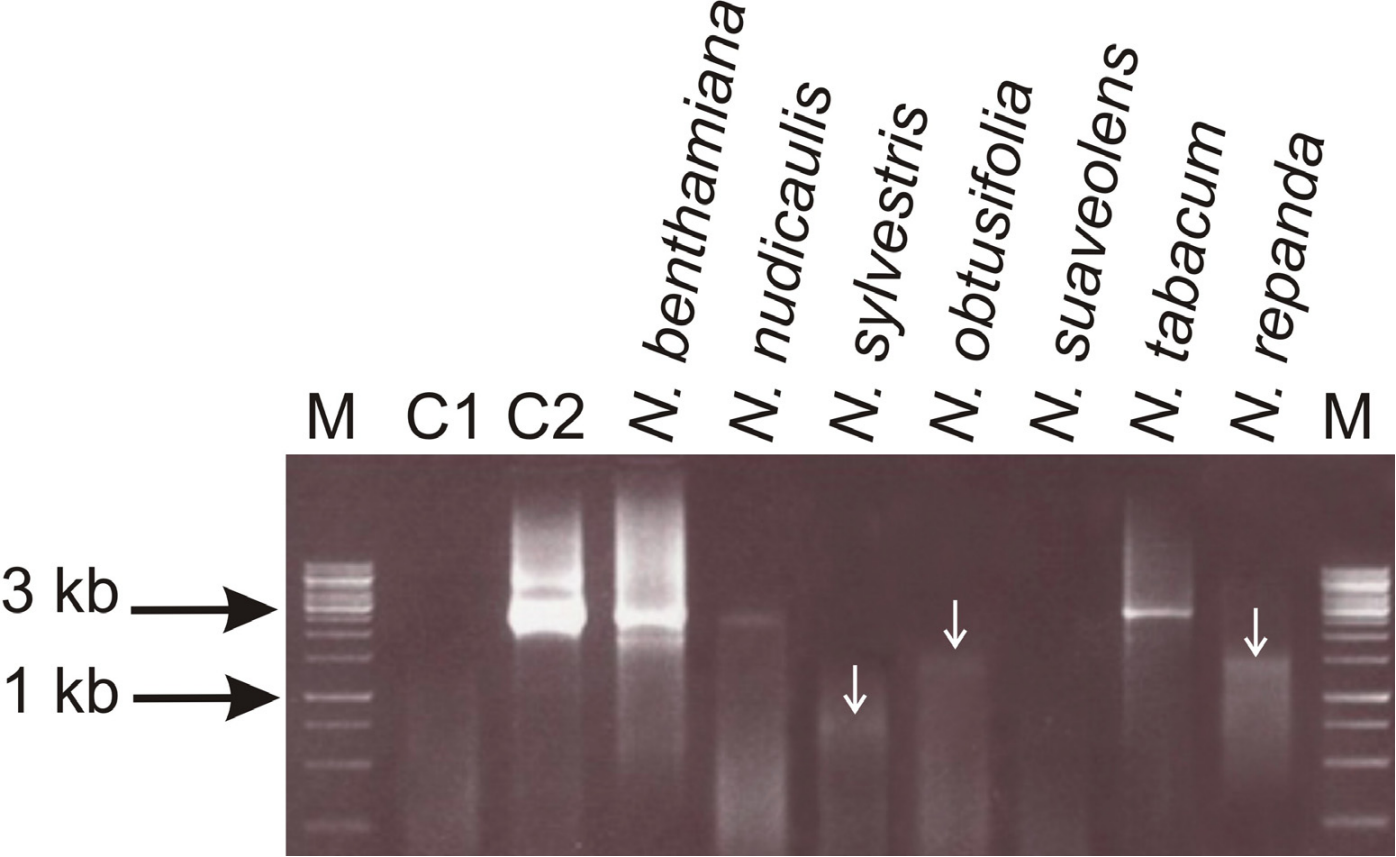
**PCR-mediated amplification of the full-length genome of CLCuMV from inoculated *Nicotiana *plants**. The ethidium bromide-stained agarose gel was photographed under UV illumination. The samples loaded on the gel resulted from PCR reactions with primer pair BegomoF/BegomoR and DNA extracted from the leaves of plants inoculated with CLCuMV (as indicated above each well). The samples in lanes C1 and C2 resulted from PCR reactions with DNA extracted from a healthy *N. benthamiana *plant and the plasmid containing the full-length genome of CLCuMV, respectively. Possible sub-genomic virus fragments are highlighted with white arrows. A DNA size marker was electrophoresed in lanes M. The sizes (bp) of selected marker bands are indicated on the left.

PCR mediated amplification with primers designed to amplify the whole CLCuMV genome (BegomoF/BegomoR) showed the presence of full-length viral genomic DNA in *N. benthamiana, N. tabacum *and *N. nudicaulis *(Figure 4). Additionally, less than full-length, subgenomic molecules were detected in *N. benthamiana, N. sylvestris, N. obtusifolia *and *N. repanda*.

**Figure 5:**
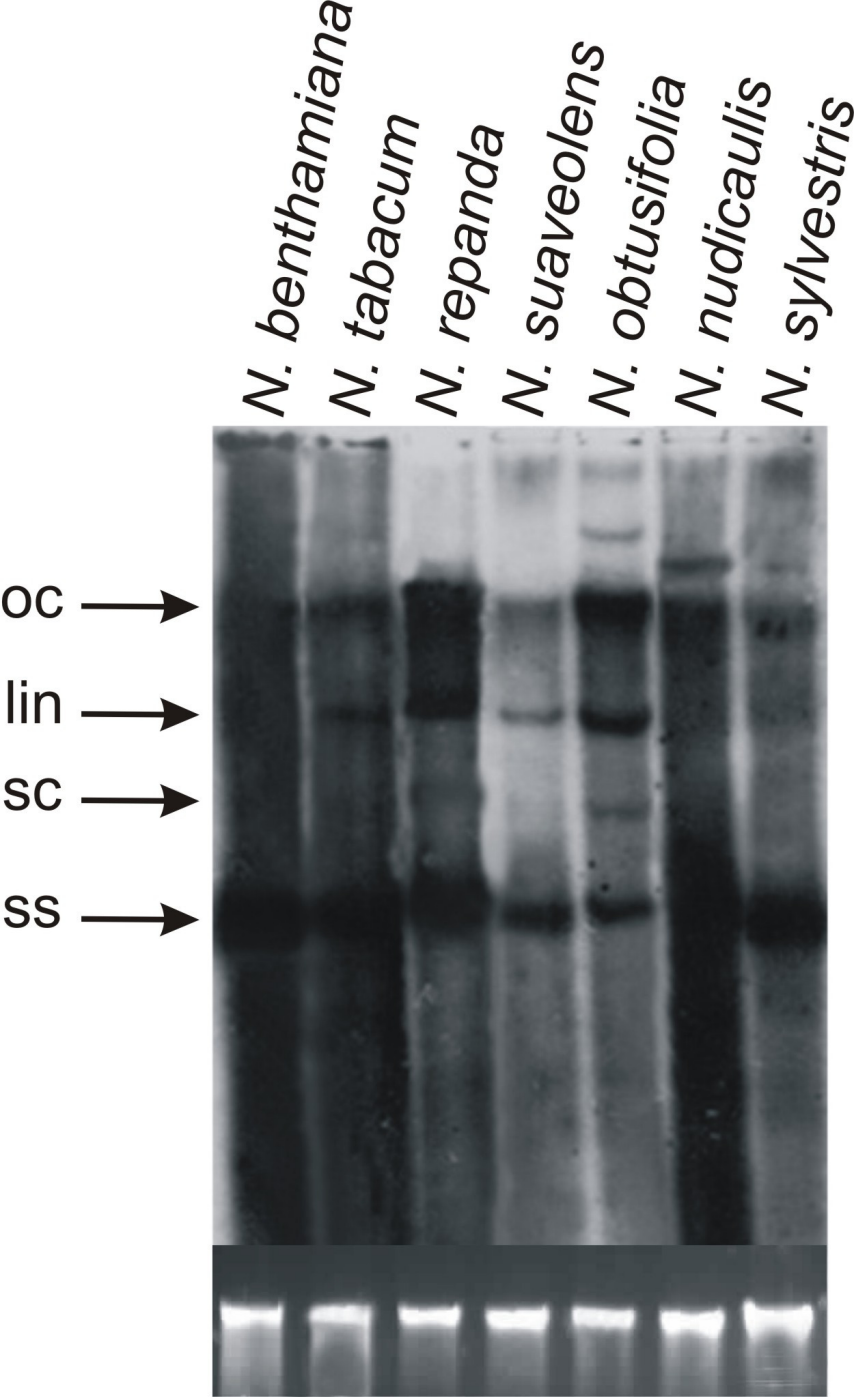
**Southern blot detection of CLCuMV in the inoculated tissues of *Nicotiana *plants**. The DNA samples loaded on the gel were extracted from leaves of plants (as indicated above each well) inoculated with CLCuMV. The leaves sampled were those inoculated with CLCuMV and were sampled at 25 dpi. An approximately equal amount of DNA (10 μg) was loaded in each case. The blot was probed with DIG-labeled CLCuMV fragment. The positions of replicative forms of viral DNA are indicated as open circular (oc), linear (lin), super coiled (sc) and single stranded (ss). A photograph of the genomic DNA bands on the ethidium bromide stained agarose gel to confirm equal loading is shown at the base.

Southern blot analysis of DNA extracted from inoculated leaves showed that there was efficient virus DNA replication in all *Nicotiana *species. The amount of single-stranded DNA was highest in *N. benthamiana *and lowest in *N. suaveolens *and *N. obtusifolia*. Several species that did not show systemic symptoms or movement accumulated higher levels of the linear form of viral DNA (Figure 5). This indicates that the lack of symptoms in all *Nicotiana *spp., except *N. benthamiana*, was due to a lack of (efficient) virus movement from the site of inoculation, rather than a lack of viral DNA replication. However, for *N. tabacum*, this was not the case with both virus replication and movement occurring without producing symptoms. Additionally, the Southern blot showed the presence of sub-genomic (defective) viral DNA forms in both *N. benthamiana *and *N. tabacum*.

### Infectivity of *Tomato leaf curl New Delhi virus *to *Nicotiana *species

**Figure 6:**
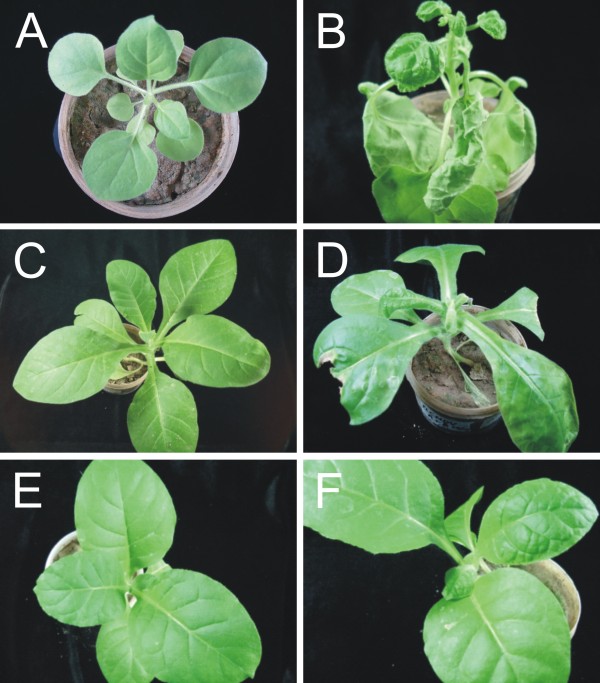
**Symptoms exhibited by three *Nicotiana *species infected with ToLCNDV**. Photographs of healthy *N. benthamiana *plant **(**A), *N. sylvestris *(C) and *N. tabacum *(E) as well as *N. benthamiana *plant (B), *N. sylvestris *(D) and *N. tabacum *(F) plants infected with ToLCNDV. Pictures were taken at 21 dpi.

**Figure 7:**
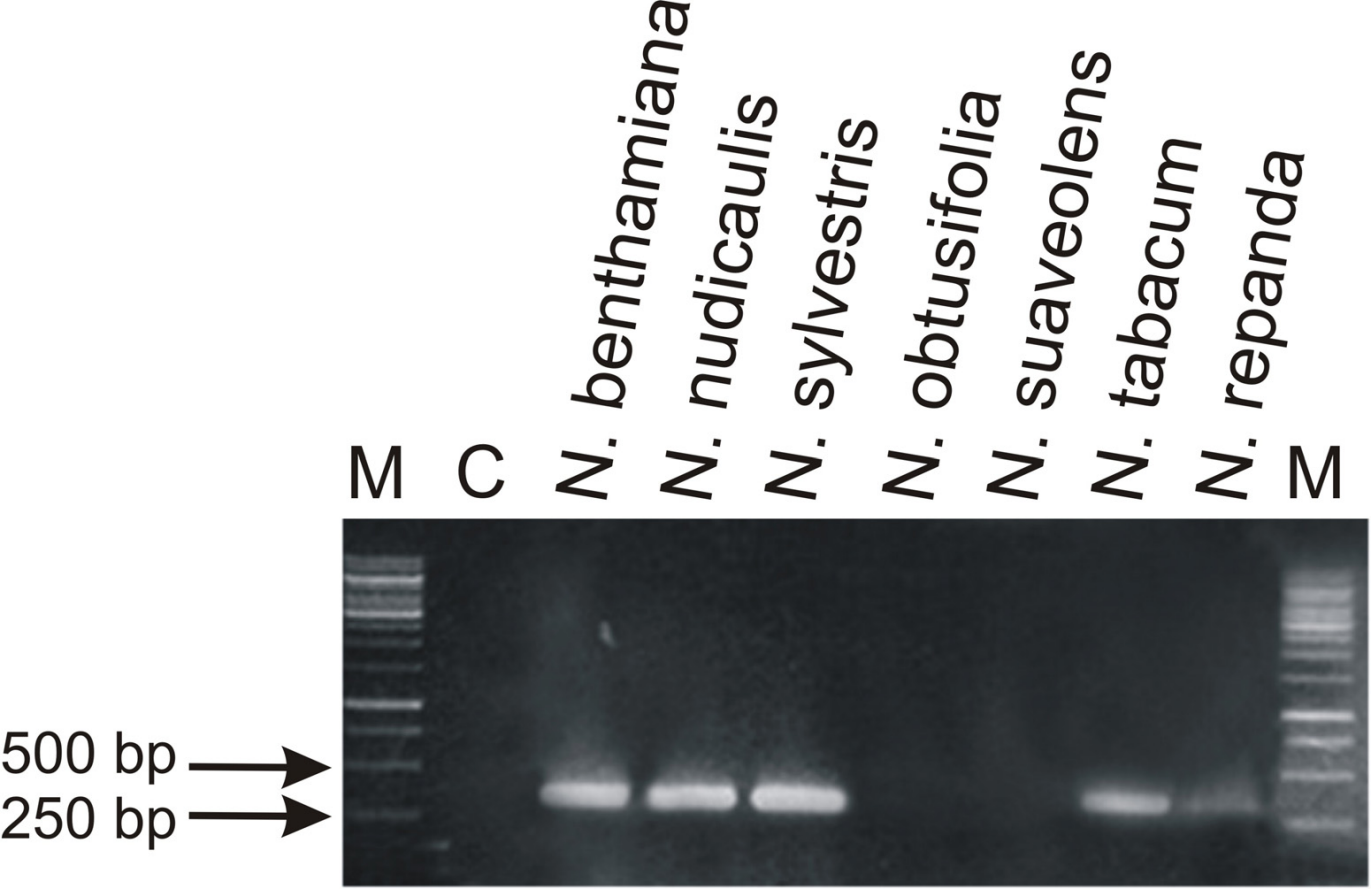
**PCR-mediated detection of ToLCNDV in inoculated *Nicotiana *plants**. The ethidium bromide-stained agarose gel was photographed under UV illumination. The samples loaded on the gel resulted from PCR reactions with primer pair ToLCNDV2F/ToLCNDV2R and DNA extracted from the leaves of plants (as indicated above each well) inoculated with ToLCNDV. The leaves sampled were developing at the time of, or developed after, inoculation and were sampled at 25 dpi. The presence of a 352 bp band indicates the systemic movement of ToLCNDV from the site of inoculation. The sample in lane C resulted from PCR amplification with DNA extracted from a healthy *N. benthamiana *plant. A DNA size marker was electrophoresed in lane M. The sizes (bp) of selected marker bands are indicated on the left.

Inoculation of *Nicotiana *species with *Tomato leaf curl New Delhi virus *(ToLCNDV) induced leaf curling and stunting symptoms in *N. benthamiana, N. tabacum *and *N. sylvestris *at three weeks post inoculation (Figure 6; Table 1). PCR-mediated detection of the virus with primers ToLCNDV2F/ToLCNDV2R in upper, non-inoculated leaves showed the presence of viral DNA in all *Nicotiana *spp. except *N. obtusifolia *and *N. suaveolens *(Figure 7). A slightly lower level of amplification product was detected in *N. repanda *than the other species, suggesting that the virus titre in this species may be lower.

**Figure 8:**
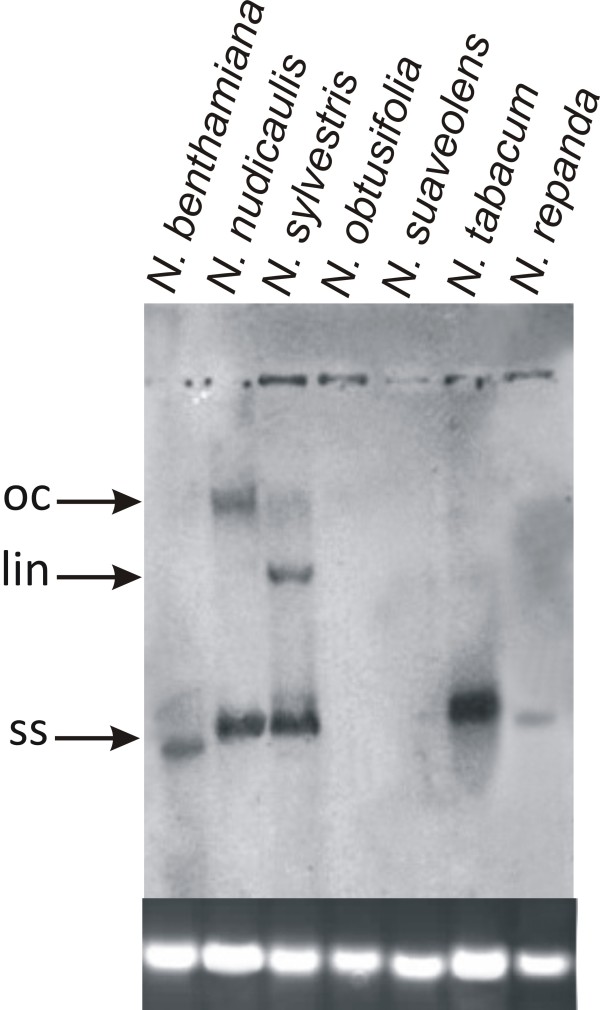
**Southern blot detection of ToLCNDV in inoculated *Nicotiana *plants**. The DNA samples loaded on the gel were extracted from leaves of plants (as indicated above each well) inoculated with ToLCNDV. The leaves sampled were developing at the time of, or developed after, inoculation and were sampled at 25 dpi. An approximately equal amount of DNA (10 μg) was loaded in each case. The blot was probed with DIG-labeled ToLCNDV fragment. The positions of replicative forms of viral DNA are indicated as open circular (oc), linear (lin) and single stranded (ss). A photograph of the genomic DNA bands on the ethidium bromide stained agarose gel to confirm equal loading is shown at the base.

Southern blot analysis showed high virus DNA levels in *N. benthamiana, N. nudicaulis, N. sylvestris *and *N. tabacum *(Figure 8). For *N. repanda *only low levels of viral DNA was detected which consisted almost entirely of ssDNA. No hybridizing bands were detected for *N. obtusifolia *and *N. suaveolens*, indicating that ToLCNDV was not able to spread from the site of inoculation (Table 2).

### PCR for RDR1m

**Figure 9:**
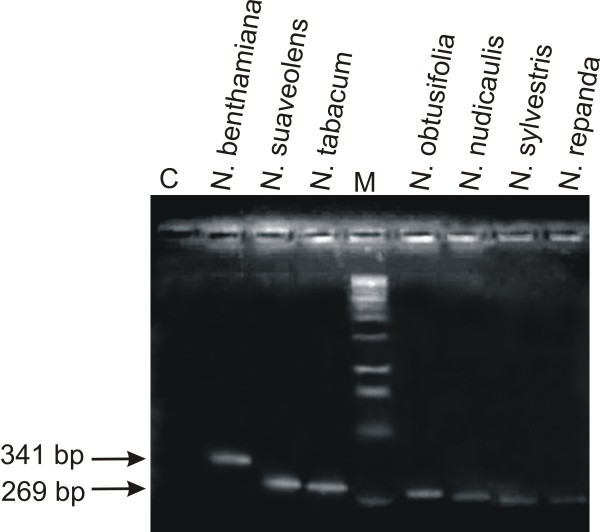
**Identification of mutated RDR1 in *Nicotiana *species**. Shown is an ethidium bromide-stained agarose gel photographed under UV illumination. Samples loaded resulted from PCR reactions using primer pair RDRf/RDRr and DNA extracted from leaves of the *Nicotiana *species. The sample in lane C resulted from a PCR reaction with primers RDRf/RDRr but lacking template DNA. A DNA size marker was electrophoresed in lane M. The sizes (bp) of selected marker bands are indicated on the left.

A natural mutation of RNA dependent RNA polymerase 1 (RDR1m), an important component of RNA silencing pathways in plants, has been identified in *N. benthamiana *and is believed to be responsible for the enhanced susceptibility of this species to numerous viruses, including geminiviruses [15]. The mutation of RDR1 in *N. benthamiana *is due to a 72 bp insertion in the gene. PCR mediated amplification across the insertion showed that only *N. benthamiana *contains this mutation (fragment size ~341 bp), with all other *Nicotiana *spp. investigated produced a PCR product approximately the size of an RDR1 lacking the insertion (~269bp; Figure 9).

## Discussion

The data presented here show that individual *Nicotiana *species differ in their susceptibility to the selected monopartite and bipartite begomoviruses. A larger number of *Nicotiana *spp. were susceptible to ToLCNDV infection and exhibited symptoms of infection. Three out of seven species were identified as susceptible with the virus inducing symptoms. *N. sylvestris *was found to be a highly susceptible host for this virus, suggesting that this species might be used as an experimental plant for this virus. The susceptibility of *N. sylvestris *(diploid species) to ToLCNDV and resistance response of *N. obtusifolia *(diploid species) to both viruses indicates that there is no clear relationship between ploidy level and susceptibility. *N. suaveolens*, a tetraploid in section *Suaveolentes *appears to be resistant to both viruses.

Monopartite begomoviruses tend to be limited to tissues immediately surrounding the phloem, whereas many bipartite begomoviruses may also infect additional tissues. This is attributed to the more efficient virus movement proteins encoded by the DNA B component of bipartite viruses [16]. This more efficient virus spread in plants may explain the wider host range of ToLCNDV in *Nicotiana *spp. identified here in comparison to CLCuMV. Some strains of ToLCNDV are mechanically transmissible, indicating that the virus is able to efficiently move from inoculated (epidermal) cells to the phloem to spread throughout the plant. Biolistic inoculation is always less efficient for CLCuMV than for ToLCNDV, which is again attributed to the inability of CLCuMV to establish infection unless introduced into phloem associated cells [13].

The ability of CLCuMV to systemically infect *N. tabacum *without causing symptoms is of interest and suggests that the virus is either unable to invade cells that are involved in symptoms or is unable to interact with factors involved in inducing symptoms. In many cases these factors are believed to be involved in the miRNA pathway, which is affected by virus pathogenicity determinants [17].

Detectable levels of defective genomic DNAs (so called defective interfering DNAs which are less than genome length) were produced in some species (*N. benthamiana*, *N. sylvestris, N. repanda and N. obtusifolia*) upon CLCuMV infection, suggesting that the virus is not well adapted to these species leading to errors in replication. Various hypotheses have been put forward regarding the mechanism(s) of production of defective geminiviral DNAs [18]. These might be produced by "jumping" of the DNA polymerase during the conversion of ssDNA to dsDNA, or during rolling circle replication of ssDNA due to the recognition of pseudo-origins of replication. Lee *et al*. [19] found that a large amount of sub-genomic viral DNAs were produced in an *Arabidopsis thaliana *ecotype (Pr-0) susceptible to *Beet severe curly top virus *(a geminivirus of the genus *Curtovirus*) but resistant to *Beet curly top virus *(a related curtovirus), suggesting that the host's resistance status may play a part in the production of defective DNAs. CLCuMV is a virus that is adapted to infect plants of the family *Malvaceae*. It is thus possible that CLCuMV is not well adapted to *Nicotiana *spp. and is thus not able to efficiently overcome host defenses, leading, by unknown mechanisms, to the production of defective DNAs. Alternatively, MacDowell *et al*. [20] suggested high levels of virus replication may be responsible for high levels of defective DNAs due to enhanced intramolecular recombination. This is consistent with our observation of the occurrence of sub-genomic, defective CLCuMV DNAs in *N. benthamiana *- a species in which virus replication is high.

*N. benthamiana *belongs to the section *Suaveolentes *of the genus *Nicotiana*. All polyploid *Nicotiana *outside this section are 2n = 4x = 48. However, for polyploid section *Suaveolentes *chromosome numbers vary between species (2n = 4x = 32 to 48) [21]. *N. sylvestris *is considered to be the most closely related extant species to the maternal progenitor of section *Suaveolentes *[22]. PCR result showed that the mutation in RDR1 is absent in *N. sylvestris*, which indicates either that the mutation is carried by the unknown paternal progenitor, or that the locus has been deleted subsequent to polyploidisation. With the exception of *N. benthamiana*, all other species assessed were either not susceptible to CLCuMV or supported asymptomatic infection. This may suggest that RDR1 is important in resisting symptomatic infection by this virus species.

The work presented here will pave the way to a more detailed analysis of the susceptibility of *Nicotiana *spp. to determine which host-encoded factors mediate resistance, for example by silencing RDR1 in *Nicotiana *spp. This information will be invaluable in future efforts to engineer virus resistance in crop plants.

## Conclusions

The results presented here show that there is no clear relationship between begomovirus susceptibility/resistance and the ploidy level of *Nicotiana *spp. This suggests that other factors, such as the presence of a fully functional RNA silencing response plays a part in this. For CLCuMV, lack of infectivity of a number of *Nicotiana *species is due to impaired movement from the site of inoculation, rather than impaired DNA replication, suggesting that these species are able to contain the virus at the initial site of entry. The insertion mutation of RDR1 was shown to be present in only *N. benthamiana *suggesting that, as first suggested by Yang *et al*. [15], the susceptibility of this species may be due to an impaired RNA silencing response. Defective viral DNAs were observed in both resistant and susceptible *Nicotiana *spp. indicating that their production is not exclusive characteristic of resistant plants.

## Materials and methods

### Origin of *Nicotiana *seed

Seed of *Nicotiana *species were kindly provided by Prof. Andrew Leitch, Queen Mary, University of London, UK and sown in pots in an insect-free containment glasshouse.

### Inoculation of plants with CLCuMV and ToLCNDV

Infectious clones of CLCuMV and ToLCNDV were transformed in competent cells of *Agrobacterium tumefaciens *strain GV3101 and inoculum was prepared for infectivity analysis. 1.5 to 2 ml inoculum having O.D. value of 1 was injected in two to three leaves per plant using a syringe. Four plants of each species were used for each treatment. Plants were kept in a containment glasshouse at 25-28°C.

### Extraction of total nucleic acids from plants and PCR

Total genomic DNA was extracted from leaf samples using the CTAB DNA method [23]. Genomic DNA was quantified by spectrophotometry and dilutions were made for PCR reaction to detect the virus.

### Detection of viral DNA in plants by Southern blot hybridization

Virus replication in inoculated and systemic (leaves developing at the time of, or subsequent to, inoculation) leaves was assessed by Southern blot hybridization. A 1.1 kb PCR fragment amplified using primers CLCV1 and CLCV2 and labeled with DIG using a PCR DIG Probe Synthesis Kit (Roche, Germany) was used as probe to detect CLCuMV. ToLCNDV was detected by using a PCR-derived DNA A fragment of 642 bp, produced with primers TLCNDV1 and TLCNDV2. The sequences of primers used are given in Table 3. Primers RDRf and RDRr were designed based upon the sequence of *N. benthamiana *RDR1m available in the nucleotide sequence databases [15].

**Table 3:** Oligonucleotide primers used in this study

Primer	Sequence	Predicted size of amplification product (base pairs)
RDRf	CGAGCCAGTTGCGGGATAATTC	269, 341
RDRr	GAGCAAAGTCAGCAGATATT	
CLCV1	CCGTGCTGCTGCCCCCATTGTCCGCGTCAC	1102
CLCV2	CTGCCACAACCATGGATTCACGCACAGGG	
PK3AV2F	AAATATCGATATGTGGGATCCACTATTAAACG	311
PK3AV2R	TCTGGTCGACCTATACATGGGCCTGTTTGT	
BegomoF	ACGCGTGCCGTGYTGCTGCCCCATTGTCC	_~_2800
BegomoR	ACGCGTATGGGCTGYCGAAGTTSASACG	
ToLCNDV2F	GTCGACAAACATGTGGGATCCATTATTGC	352
ToLCNDV2R	ATCGATCTTCTATACATTCTGTACATTC	
TLCNDV1	GCAGATATCATCATTTCAACGC	642
TLCNDV2	CATACTTGCCGGCCTCTTGTTG	

## Competing interests

The authors declare that they have no competing interests.

## Authors' contributions

SA performed the experiments and prepared manuscript. SM provided overall directions regarding the designing of all experiments, writing and supervised the work. RWB was involved in critical review of the work and writing the manuscript. The final manuscript was read and approved by all authors.
